# Biopsychosocial correlates of body satisfaction in 7- to 8-year old children: a cross-sectional and prospective investigation

**DOI:** 10.1186/s40337-024-01178-7

**Published:** 2024-12-30

**Authors:** Danielle L. Smith, Stephanie R. Damiano, Siân A. McLean, Eleanor H. Wertheim, Susan J. Paxton

**Affiliations:** School of Psychology and Public Health, La Trobe University, Melbourne, Australia

**Keywords:** Body satisfaction, Biopsychosocial model, Biological, Psychological, Sociocultural, Risk factors, Children

## Abstract

**Background:**

Biopsychosocial factors have been associated with body satisfaction/dissatisfaction and related body image concerns in adolescence; however, few studies have investigated these relationships in middle childhood, an important developmental phase for body satisfaction. This study investigated relationships between a range of biological (body mass index), psychological (child anxiety/depression, self-esteem, and self-oriented and socially prescribed perfectionism) and sociocultural (mother’s body dissatisfaction and comments about child’s appearance, father’s body dissatisfaction and comments about child’s appearance, peer teasing and child’s media exposure) factors and body satisfaction cross-sectionally and longitudinally in a sample of 7- and 8-year-old children.

**Methods:**

In this study, participants from the longitudinal Children’s Body Image Development Study (in which children had been followed-up annually from 3 years old) were assessed by interview at 7 years old (Time 1; *n* = 293: girls = 167, boys = 126) and 8 years old (Time 2; *n* = 222; girls = 126, boys = 96) and their parents completed a questionnaire at each time point.

**Results:**

Multiple regression analyses revealed that child self-esteem, socially prescribed perfectionism, and mother body dissatisfaction in the total sample at 7-years, as well as child self-esteem and mother body dissatisfaction in the total sample at 8-years were significant unique cross-sectional correlates of child body satisfaction. While self-esteem outcomes were replicated at both time points for boys and girls, some differences in patterns were found for other variables in the subsamples of boys versus girls across time points. Prospectively in partial correlations (controlling for Time 1 child body satisfaction), mother’s body dissatisfaction predicted later child body satisfaction in boys and child self-esteem predicted later body satisfaction in girls. However, no longitudinal biopsychosocial predictors were identified as contributing unique variance in child body satisfaction from 7- to 8-years old after accounting for Time 1 (7-yearold) child body satisfaction.

**Conclusions:**

These findings point to important psychosocial factors that are consistently related to body satisfaction in children and could be targets for intervention, but also suggest that a number of biopsychosocial variables develop concurrently with body satisfaction.

## Abstract (plain language summary)

A growing body of evidence suggests that children develop body image attitudes as young as 3 years old and that dissatisfaction with one’s own body increases in prevalence at around 6 years old. Body dissatisfaction is consistently associated with physical and psychological consequences, including clinical eating disorders. Despite this, there are few studies identifying factors associated with body dissatisfaction involving children. This knowledge is crucial for accurate and effective prevention and intervention strategies to be developed for this population prior to the onset of adverse physical and psychological outcomes. The current study performed a cross-sectional and prospective investigation of relationships between biological, psychological, and sociocultural factors and body satisfaction in 7- and 8-year-old boys and girls to identify possible risk factors. Overall, child self-esteem and socially prescribed perfectionism as well as body dissatisfaction in mothers were the key factors related to children’s body satisfaction suggesting these as possible targets for prevention and intervention.

## Background

Recent research suggests that the formation of many body image attitudes occurs during early childhood. Children as young as 3 years old demonstrate an awareness of societal preferences for thinness [1, 2] and by 6 years old an increasing proportion of children are dissatisfied with their body [3]. Studies have indicated that body image concerns are largely stabilised by 11 to 12 years old [4, 5]. This is very concerning given the increased risk of psychological problems associated with body dissatisfaction and subsequent engagement in body change strategies, including clinical eating disorders [6].

The biopsychosocial model of body image development [7–9] proposes that biological, psychological, and sociocultural factors influence the development of body satisfaction and dissatisfaction. Some studies have investigated relationships between biopsychosocial factors and a range of body image concerns in children [10–13]; however, research involving children aged 8 years and younger, particularly prospective research, is sparse. Crucially, few single studies have investigated a range of biopsychosocial factors in combination in this population to identify the most relevant risk factors in this age group, although there are several studies that have done so in adolescent and preadolescent samples [14–16]. The present research aims to investigate cross-sectional and prospective relationships between selected biopsychosocial variables and body satisfaction/dissatisfaction in 7- and 8-year-old girls and boys to expand our understanding of the risk and protective factors for body satisfaction.

### Biological variables and body satisfaction

Pertaining to biological variables, body mass index (BMI) is the leading risk factor investigated in research on the development of body satisfaction/dissatisfaction [9]. It is proposed that body dissatisfaction develops as a result of the perception that one’s body size is markedly discrepant from societal ideals [9] which may be reinforced by appearance-related teasing [17]. Therefore, although BMI is conceptualised as a biological construct within etiological models, this hypothesis suggests a cognitive element underpinning the relationship between body size and body dissatisfaction. Significant relationships between higher BMI and greater body dissatisfaction have been identified in some cross-sectional and longitudinal studies involving children aged between 5 and 9 years old [18, 19], but not others [12]. Given the inconsistent findings from this research, the role of BMI in children’s body satisfaction is not well understood and further studies involving this population are needed.

### Psychological variables and body satisfaction

Anxious/depressed affect, self-esteem, and perfectionism are among the psychological constructs most studied within the biopsychosocial model [9]. Anxious/depressed affect is proposed to increase an individual’s perception that one’s own body is discrepant from the societal ideal leading to body dissatisfaction [20, 21]. Despite studies demonstrating cross-sectional and longitudinal relationships between anxious/depressed affect and body dissatisfaction in adolescents and preadolescents [16, 22], very limited research has included children younger than 8 years. Among these studies, Nichols et al. found very few cross-sectional and longitudinal relationships between anxious/depressed affect and body dissatisfaction in an earlier examination of the present sample, and of the significant relationships found, these were generally small [12]. More recently, Bufferd et al. found that diagnoses of anxiety disorders at age 3 and depression at age 6 were prospective predictors of greater body dissatisfaction at age 12 [23].

Self-esteem refers to the subjective evaluation of oneself and encompasses beliefs about one’s own attributes and capabilities and associated emotional states [24]. A lower level of regard for one’s own worth is proposed to increase an individual’s vulnerability to internalise societal appearance ideals, engage in upward appearance comparisons with others, and consequently, become dissatisfied with one’s own body [8]. Research investigating relationships between self-esteem and body satisfaction consistently shows a positive cross-sectional association in adolescents [16, 25] and, in fewer studies, preadolescents [26, 27] and young children [12]. However, prospective studies involving adolescents have produced inconsistent findings regarding the direction of relationships between self-esteem and body satisfaction [28–30] and the very few studies involving preadolescents and young children have not shown prospective relationships between self-esteem and body satisfaction [11, 12]. In light of empirical evidence, which indicates a decline in self-esteem at around 8 years old [31, 32], this appears a crucial age to further examine its relationship with body satisfaction.

Perfectionism is typically defined by the tendency to engage in setting and striving to attain unrealistic standards, rigid adherence to high standards, selective attention to and overgeneralisation of failure, and overly critical self-evaluation in terms of ability to achieve standards [33]. Flett, Hewitt and De Rosa have conceptualised distinct domains of perfectionism, including *self-oriented perfectionism* (i.e., an intrapersonal dimension that involves the self-imposed expectation of perfection) and *socially prescribed perfectionism* (i.e., an interpersonal dimension involving the perception that others are demanding perfection from oneself) [34]. The key features of perfectionism are theorised to increase an individual’s vulnerability to apply unattainable standards to their appearance, thus leading to body dissatisfaction [35]. Studies support cross-sectional relationships between subtypes of perfectionism and body dissatisfaction in adolescents [25, 36] and some studies have shown significant relationships between these variables in preadolescents [37, 38]. Prospective studies, however, have shown inconsistent findings in these populations [39, 40]. Few studies have investigated the role of perfectionism in young children’s body image; however, these studies show promising evidence for including perfectionism in etiological models for this population, and a higher level of socially prescribed perfectionism appears to be particularly related to greater body dissatisfaction [12, 13]. Given the lack of studies investigating perfectionism as a risk factor for body dissatisfaction particularly in children, further studies are required to validate existing findings.

### Sociocultural variables and body satisfaction

Parents, peers, and media are identified as the three predominant contexts of communication of societal appearance ideals [8, 9, 41]. In the context of social learning theory [42], parental attitudes towards their child’s shape, weight, and eating behaviours expressed through teasing, criticism, and encouragement to lose or control weight (i.e., direct influences) are suggested to influence body image by placing value on appearance [41]. Parents are also proposed to influence the development of their child’s body dissatisfaction by expressing negative attitudes towards their own body (i.e., indirect influences) [43]. Persistent exposure to direct and/or indirect appearance pressures from parents is proposed to increase the extent to which a child adopts societal appearance ideals and the tendency to engage in upward appearance comparisons, consequently leading to body dissatisfaction [44].

Many cross-sectional and prospective studies have found evidence for the role of direct parental influences (e.g., weight-related teasing) in body dissatisfaction in adolescents [45, 46] and a small number of studies support these relationships in children as young as 3 years old [47–49]. There are fewer studies that investigate relationships between indirect parental influences and body image outcomes including body satisfaction in adolescents [28, 30, 50] and inconsistent findings have been found in samples of young children [47, 51, 52]. In addition, most studies involving young children have explored the role of mothers only. Fathers may also play a distinct role in their children’s body image development [53]. An additional gap in the literature is that parental factors have rarely been investigated prospectively in studies of young children. These issues will be addressed by the current research in relation to body satisfaction.

The peer environment provides further opportunities for learning about societal norms and expectations including those pertaining to appearance [54]. Similar to parents, peers are suggested to directly influence body satisfaction in several ways including through teasing about weight and appearance [4, 22] and engaging in conversations about body size and shape, physical characteristics, dieting, and exercise [55, 56]. Peers are also suggested to indirectly influence body satisfaction development through modelling of their own attitudes and behaviours, including body dissatisfaction and dieting behaviours [8].

Meta-analyses have provided support for cross-sectional relationships between peer teasing and body dissatisfaction in both children and adolescents; however, it is less clear whether teasing is a prospective risk factor for body dissatisfaction as research findings are inconsistent [57–59]. A small number of studies has investigated relationships between peer teasing and body satisfaction in young children. Some cross-sectional studies indicate that a greater frequency of appearance teasing from peers is significantly related to heightened body dissatisfaction in both boys and girls as young as 8 years old [60, 61]. The current research will expand on existing research by investigating relationships between peer teasing and body dissatisfaction in children in the context of a biopsychosocial model.

Exposure to media depicting idealised body shapes and sizes has also been proposed to influence body satisfaction [8]. According to social learning theory [42], repeated exposure to appearance ideals through media increases an individual’s vulnerability to accept these idealized portrayals as reality and perceive that outward appearance is central to self-worth. As idealized media portrayals are mostly unattainable, endorsing these as normative may lead to a perceived discrepancy between one’s own body and that of models, and consequently dissatisfaction with one’s body [62]. In a meta-analysis by Huang, Peng, and Ahn in which relationships between appearance-based media exposure and body satisfaction were investigated, media exposure showed moderate to large effects on body satisfaction in adolescents, while effect sizes were smaller for children and adults [63]. There are very few longitudinal studies exploring the relationship between media exposure and body satisfaction in children. However, cross-sectional studies involving 5- to 8-year-old girls have identified media exposure as a significant correlate of body image outcomes [10, 19].

### Relative influence of biopsychosocial factors on body satisfaction

Very few studies have investigated a range of biological, psychological, and sociocultural variables in combination, and most of these are cross-sectional studies involving adolescent samples (e.g., 7,14,16). One prospective study involving adolescent girls investigated relationships between biopsychosocial variables and body dissatisfaction over a 1-year period [40]. The study suggests that some biopsychosocial variables may be stronger risk factors than others for adolescents, particularly peer teasing and self-esteem, and indicates the importance of investigating a wide range of factors simultaneously in order to identify the most important targets for prevention and intervention programs. There are currently no studies that investigate relationships between a combination of biopsychosocial factors and body satisfaction/dissatisfaction in children younger than 8 years old. Importantly, biological, psychological, and sociocultural factors have been shown to be individually related to body satisfaction in this population. However, identifying which factors are the most relevant in the development of body satisfaction in children is essential to guide the best variables to target for prevention.

### Study aims and hypotheses

The goal of the current study was to gain further understanding of the key relationships between body satisfaction and biological (BMI), psychological (anxious/depressed affect, socially prescribed perfectionism and self-oriented perfectionism, and self-esteem), and sociocultural (mother and father body dissatisfaction, mother and father comments about weight and food, peer teasing and child media exposure) factors in 7- and 8-year-old girls and boys. This age span is important to investigate for a number of reasons in addition to the fact that it has been a neglected one in the body satisfaction literature as highlighted above. Cognitively, up until about 6–7 years old, children typically engage in overestimated self-evaluations of their competence and qualities due to an inability to assess themselves in comparison to others. However, around 8-years-old children become more able to compare themselves to others for the purpose of self-evaluation, and to discriminate between actual and ideal attributes [31, 32]. In addition, at this time, children are exposed to more negative feedback from teachers, parents, and peers [64]. Such changes in social environment combined with the capacity to engage in social comparisons contributes to children making more accurate self-evaluations and may promote a decline in self-esteem and related self-assessments [64]. Body satisfaction may be one attribute affected by these changes.

The first aim was to examine cross-sectional relationships between body satisfaction and biopsychosocial variables in 7- and 8-year-old girls and boys. Based on the empirical research described above, it was hypothesised that BMI, self-esteem, parent body dissatisfaction, parent direct comments about their child’s appearance, media, and peer teasing would emerge as uniquely contributing to variability in body satisfaction, when considered in combination. Other variables proposed by the biopsychosocial model were explored but were not anticipated to be unique correlates. In addition, relationships between biopsychosocial variables and body satisfaction will be examined in boys, to extend existing research which has predominantly involved girls.

The second aim was to examine which biopsychosocial variables assessed at age 7 prospectively predicted body satisfaction at 8-years-old. Based on empirical research described above, it was hypothesised that anxiety/depression, socially prescribed perfectionism, parent body dissatisfaction, parent direct comments about their child’s appearance, and peer teasing would emerge as uniquely contributing to change in body satisfaction when considered together.

## Method

### Participants

Children were recruited in Melbourne, Australia, as part of the longitudinal Children’s Body Image Development Study (CBIDS) [2]. Recruitment strategies included advertisements in childcare centres and playgroups, radio advertisements, and social media campaigns. Children entered the study at age 3 years and a second cohort of children entered the study at age 5 years, to participate in annual interviews. The current study used data collected when children were aged 7 years (Time 1) and 8 years (Time 2).

At Time 1, participants were 293 children, *n* = 167 girls *M*_age_ = 7.45 years (*SD* = 0.33), *n =* 126 boys *M*_age_ = 7.50 years (*SD* = 0.33), *n =* 282 mothers *M*_age_ = 41.65 years (*SD* = 4.52, range = 27 to 56 years), and *n* = 176 fathers *M*_age_ = 43.69 years (*SD* = 5.30, range = 33 to 64 years). At Time 2, participants were 222 children, *n* = 126 girls *M*_age_ = 8.44 years (*SD* = 0.34), *n =* 96 boys *M*_age_ = 8.45 years (*SD* = 0.38), *n =* 208 mothers *M*_age_ = 43.02 (*SD* = 4.66, range = 32 to 59 years), and *n* = 147 fathers *M*_age_ = 44.45 (*SD* = 5.31, range = 34 to 65 years).

## Measures

### Demographic data

At Time 1 and 2, parents provided data on their date of birth, level of education, occupation, employment status, and primary caregiver status. Parents also provided data on their child’s sex, date of birth, and ethnic background. Height and weight of children were measured by researchers during an interview at Time 1 and 2, and BMI-for-age z-scores were calculated using syntax developed by the World Health Organisation [65].

Demographic data collected from parents at Time 1 indicated that the majority of primary caregivers were female (95.4%). The majority of families involved resided in high (65%) and average (30%) socioeconomic areas relative to Australian population data, and a further 5% resided in disadvantaged socioeconomic areas [65]. At Time 1, most children were reported to be of Australian heritage (46%), 23% were of Anglo-Saxon heritage, 10% were of European, Middle Eastern, or Asian heritage, and the remaining 21% reported being of multiple heritages. Overall, the present sample came from more culturally diverse backgrounds compared to Australian representative data, which indicates that most Australians reported English (36.1%), or Australian (33.5%) ancestry [66].

### Parent measures

#### Parent body dissatisfaction

To assess parent body dissatisfaction, the 29 items from 3 subscales of the Body Attitudes Questionnaire (BAQ) comprising feeling fat, self-disparagement, and salience of weight subscales were used [66]. Participants rated items (e.g., “I spend a lot of time thinking about my weight”) from 1 (*Strongly Disagree)* to 5 (*Strongly Agree)*. A total score was summed (possible range from 29 to 145), with higher scores indicating greater body dissatisfaction. Good internal consistency (α = 0.87) and convergent and discriminant validity have been reported for the BAQ in the original sample of 14- to 65-year-old females [67]. Internal consistency for the measure used in the present sample of mothers was Cronbach’s α (α) = 0.94 at Time 1 and α = 0.93 at Time 2, and for fathers α = 0.91 at Time 1 and α = 0.93 at Time 2.

#### Parent comments

The 6-item parent body and eating comments scale was devised for the CBIDS. Four items assessed verbally expressed concerns from parents regarding body shape/weight and eating directed toward their child or others (e.g., “I advise my child to not eat certain foods because they may put on weight”). One item assessed verbal criticism of child clothing choices from the Feedback on Physical Appearance Scale [68] and one item assessed concern about child overeating from the Child Feeding Questionnaire [69]. Items are scored from 1 *(Strongly Disagree)* to 5 (*Strongly Agree*). The scale was scored by calculating the mean of the 6 items and possible scores range from 1 to 5, with higher scores indicating parents’ greater endorsement of making body and eating related comments to their child. Psychometric data is not available in other studies. Internal consistency for the present sample of mothers was α = 0.60 at Time 1 and α = 0.63 at Time 2, and for fathers was α = 0.70 at Time 1 and α = 0.72 at Time 2, which was considered acceptable for a 6-item scale.

### Child measures

#### Child body satisfaction

A 19-item adaptation of the Body Esteem Scale (BES) measured child body satisfaction [70]. The measure was adapted from self-report to interview format to meet the language capability of young children (e.g., “I like what I look like in pictures” was rephrased to “Do you like how you look in pictures?”) and items considered conceptually challenging were removed from the scale (e.g., “I have a high opinion about the way I look”). Children responded verbally either “Yes” or “No” to items and the scale was scored by adding the number of positive responses. Total scores could range from 0 to 19, with higher scores indicating greater body satisfaction. The BES has been shown to have acceptable internal consistency and convergent validity in samples of similar age children [71, 72]. Internal consistency for the present sample was α = 0.74 at Time 1 and α = 0.76 at Time 2.

#### Child anxiety/depression symptoms

As there are no child-report measures of anxiety/depression symptoms which are psychometrically adequate for use with the age of the current sample, child anxiety/depression symptoms were assessed using the 8-item Anxiety/Depression syndrome scale of the Child Behaviour Checklist (CBCL) and was completed by the child’s primary caregiver [73]. The caregiver rated statements relating to their child’s tendency to display internalising symptoms over the preceding six months (e.g., “My child is overly fearful or anxious”) on a 3-point scale of 0 (*Not True)*, 1 (*Somewhat or Sometimes True*), and 2 (*Very True or Often True*). Responses are summed, computed into T-scores, and a total mean T-score is calculated. T scores less than 67 are considered in the normal range for internalising symptoms, between 67 and 70 are considered borderline clinical, and above 70 are in the clinical range. Acceptable to excellent internal consistency and convergent validity have been reported [73, 74]. In the present research, internal consistency was α = 0.80 at Time 1 and α = 0.82 at Time 2.

#### Child self-esteem

Self-esteem was measured using three items of the Global Self-Worth Scale of the Self-Perception Profile for Children (SPPC), which were adapted from self-report to interview format [75]. Interviewers presented children with a scenario (e.g., “Some kids like the kind of person they are BUT other kids often wish they were someone else”) and asked “What about you? Do you like the kind of person you are, or do you often wish you were someone else?” Interviewers also asked children whether their response was “Sort of true” or “Really true”. Items were scored on a four-point scale, where 1 indicated the lowest and 4 indicated the highest perceived level of self-worth. The mean of the three items was calculated to obtain the scale total and total mean scores ranged from 1 to 4, with higher scores indicating greater perceived global self-esteem. Good internal consistency has been reported for an adapted interview version of this scale in an Australian sample of 5- to 8-year-old girls [11]. In the present sample, Cronbach’s alphas were α = 0.72 at Time 1 and α = 0.68 at Time 2.

#### Child perfectionism

A modified 8-item version of the Child and Adolescent Perfectionism Scale (CAPS) assessed perfectionism [76, 77]. Two subscales were used for the current study comprising 4 items assessing self-oriented perfectionism (i.e., holding excessively high personal standards and intrinsic motivation to attain them) and 4 items assessing socially prescribed perfectionism (i.e., the belief that others hold excessively high standards of oneself). Items were rephrased from self-report to interview format (e.g., “I try to be perfect in everything I do” was rephrased to “Do you try to be perfect in everything you do?”). Children responded “Yes”, “No” or “Unsure/no response” to each question, and further indicated their level of certainty by stating whether their response was “Really true” or “Sort of true”. A simplified four-point response format was used, ranging from 1 (*No*,* not true*) to 4 (*Yes*,* really true*). Scores for both the SOP and SPP subscales ranged from 4 to 16, and higher scores indicated greater perfectionism in each domain. Very good internal consistency and concurrent and discriminant validity has been reported for the original measure in adolescent samples [77, 78]. Internal consistency in the present sample for SOP was Time 1 α = 0.68 at Time 2 α = 0.63, and for SPP was Time 1 α = 0.60 and Time 2 α = 0.69.

#### Peer teasing

As the planned measure of appearance teasing, the child-reported Weight-Related Teasing subscale of the Perception of Teasing Scale (POTS-WR) [79] had poor internal consistency, a parent-report version of the POTS-WR (POTS-P) was designed for the current study. The POTS-P assessed frequency with which parents perceived their child to experience weight-related teasing. Four items of the original 7 were retained (e.g., “Children tease your child because he/she is heavy”). Parents responded on a 5-point scale ranging from 1 *(Never)* to 5 *(Very Often)*. Item responses were summed and the total scale score ranged from 4 to 20, with higher scores indicating greater parent perception of teasing experienced by their child. Internal consistency for the parent-report measure used in the present research was acceptable (Time 1 α = 0.66 and Time 2 α = 0.72). A parent-report measure of teasing has not previously been used; therefore, psychometric data from other studies is not available.

#### Child media exposure

To assess children’s frequency of media exposure, an adapted version of the measure used by Dohnt and Tiggemann was designed for the current research [19]. The child’s primary caregiver was asked to estimate the daily average number of hours that their child spends watching television, DVDs, online videos (e.g., YouTube), and playing computer games excluding for school use on a usual weekday and weekend day. Media use was measured in 0.5-hour (30 min) increments and parents estimated the daily average number of hours up to 6 h, and the total hours per week was calculated. Psychometric data is not available in other studies for this measure.

### Procedure

The study was approved by the La Trobe University Science, Technology and Engineering Faculty Human Ethics Committee (FHEC10/R81). Written consent was obtained from parents for self and child to participate, and verbal assent was obtained from children prior to the interview.

Parents completed the parent questionnaire either as a hard copy or online depending on their preference. Children were interviewed individually by trained researchers either at the participant’s home or school. The interview comprised a number of measures, including distractor questions; however, only the measures relevant to the aims of this study are discussed in the manuscript. Following participation, children received a sticker and families received a $10 shopping voucher and entry into a prize draw for a shopping voucher of a higher value.

### Data preparation and analysis

Data were examined for normality. At Time 1 and Time 2, violation of normality was identified for seven variables. Therefore, to meet assumptions of parametric tests, a reflect and logarithmic transformation was performed for child body satisfaction at Time 1 and Time 2 and a logarithmic transformation was performed for child media exposure at Time 1 and Time 2. Child BMI, child anxiety/depression, child self-esteem, child socially prescribed perfectionism, and peer teasing did not meet normality requirements at Time 1 or with transformation at either Time 1 or Time 2 as these variables were substantially skewed. These variables remained untransformed and non-parametric analyses were performed.

Cross-sectional relationships between biopsychosocial variables (i.e., child BMI, child anxiety/depression, self-esteem, self-oriented perfectionism, socially prescribed perfectionism, parent body satisfaction, parent comments, peer teasing, and child media exposure) and child body satisfaction were first examined at both Time 1 and Time 2 using Pearson (for normally distributed variables) and Spearman (for non-normally distributed variables) correlation analyses for the total sample and boys and girls separately. To identify the variance in child body satisfaction accounted for by biopsychosocial factors, predictor variables that were correlated significantly (*p* < .05) with body satisfaction were entered into multiple regression analyses for the total sample and for boys and girls separately.

Prospective relationships between Time 1 biopsychosocial variables and Time 2 child body satisfaction were explored using partial Pearson (for normally distributed variables) and Spearman (for non-normally distributed variables) correlation analyses, whereby child body satisfaction at Time 1 was controlled. A hierarchical multiple regression analysis was then performed to identify variance accounted for by Time 1 biopsychosocial variables that were significantly correlated (*p* < .05) with Time 2 child body satisfaction after accounting for child body satisfaction at Time 1. For prospective analyses only, non-significant trends at the level of *p* < .10 were also included in regression analyses to ensure all relevant variables were included in analyses. Both partial correlation and hierarchical regression analyses were conducted for the total sample and for boys and girls separately.

G*Power (version 3.1.9.6) was used to perform power analyses to confirm sufficient sample sizes [80]. Sample sizes for regression analyses met the minimum number of participants recommended by Tabachnick and Fidell [81], except in some cases of sex specific analyses which are indicated in the Results section where relevant. Caution is required when interpreting findings from analyses which did not meet the required sample size. All statistical analyses were performed using SPSS version 27.0. Exact *p* values are reported, except where less than *p* = .001. All analyses used two-tailed tests of significance.

## Results

### Participant characteristics and retention

Table 1 presents descriptive data for the total sample of children at Times 1 and 2. Participant retention rate from Time 1 to Time 2 was 76%. A series of non-parametric Mann-Whitney-U tests were performed to examine whether scores for body satisfaction and biopsychosocial variables at Time 1 differed significantly for children who participated at Time 2 compared with those who did not. Few significant differences were found. Participants who were retained at Time 2 (*n* = 209, *Md* = 4.00) had significantly lower scores for peer teasing than those not retained (*n* = 66, *Md* = 5.00), *U* = 5782.50, *z* = -2.11, *p* = .03. Participants retained at Time 2 (*n* = 211, *Md* = 17.33) had significantly lower scores for media exposure than those not retained (*n* = 67, *Md* = 20.00), *U* = 5172, *z* = -3.31, *p* = .01. It was considered that these differences were unlikely to have exerted much impact on findings given the relatively high retention rate overall [82].

**Table 1:** Summary of descriptive statistics for body satisfaction and biopsychosocial variables at Times 1 and 2 for the total sample

Time 1 Variable	Mean (SD)/Median (IQR) Time 1 Time 2		
Body image outcome			
Child body satisfaction^a^	15.84 (2.44)	17.23 (2.34)	
Biological variables Child BMI	16.53 (2.73)	16.92 (2.44)	
Psychological variables			
Child anxiety/depressiona	48.06 (7.02) a	47.94 (6.15) a	
Child self-esteem^b^	4.00 (0.50)	4.00 (0.25)	
Child self-oriented perfectionism	10.35 (3.37)	9.64 (3.31)	
Child socially prescribed perfectionism^b^	7.00 (5.00) ^b^	6.00 (4.00) ^b^	
Sociocultural variables			
Mother body dissatisfaction	69.89 (17.70)	70.07 (17.04)	
Mother comments	1.90 (0.59)	1.92 (0.58)	
Father body dissatisfaction	59.10 (14.74)	59.76 (16.54)	
Father comments	2.16 (0.63)	2.16 (0.65)	
Peer teasing^b^	5.00 (2.00)^b^	5.00 (2.00) ^b^	
Child media exposure^a^	19.73 (8.49) a	21.83 (9.81) a	

Note. SD = Standard deviation, IQR = Interquartile range, ^a^ = mean reported for untransformed variables, ^b^ = median reported due to non-normal distribution of variable

### Cross-sectional relationships between biopsychosocial variables and body satisfaction at 7 and 8 years old

Bivariate correlations were conducted between biopsychosocial variables and body satisfaction at 7 and 8 years old for the total sample of children and for boys and girls separately (see Table 2), followed by the multivariate analyses (see Tables 3 and 4).

**Table 2:** Cross-sectional correlations between biopsychosocial variables and body satisfaction at Times 1 (7 years) and 2 (8 years) for the total sample, boys, and girls

	Body satisfaction					
	Total Sample		Boys		Girls	
Biopsychosocial Variable	Time 1	Time 2	Time 1	Time 2	Time 1	Time 2
Biological variables						
Child BMI a	0.02	− 0.09	− 0.06	− 0.16	0.05	− 0.06
Psychological variables						
Child anxiety/depression a	− 0.06	− 0.24**	− 0.12	− 0.22*	− 0.02	− 0.24*
Child self esteem a	0.44**	0.36**	0.42**	0.20*	0.45**	0.46**
Child self-oriented perfectionism	− 0.01	0.01	− 0.04	− 0.03	− 0.12	0.02
Child socially prescribed perfectionism a	− 0.27**	− 0.14*	− 0.24**	− 0.04	− 0.29**	− 0.21*
Sociocultural variables						
Mother body dissatisfaction	− 0.16**	− 0.16*	− 0.10	− 0.19	− 0.20*	− 0.14
Mother comments	− 0.06	− 0.06	− 0.00	0.00	− 0.09	− 0.13
Father body dissatisfaction	0.01	− 0.07	0.17	0.06	− 0.11	− 0.13
Father comments	0.06	− 0.09	0.10	− 0.14	0.03	− 0.08
Peer teasing a	− 0.19**	− 0.13	− 0.18*	0.12	− 0.20*	− 0.15
Child media exposure	− 0.11	− 0.03	0.01	0.01	− 0.20*	− 0.06

*Note.* ^a^ Spearman’s rho statistic used due to non-normal distribution of scores. *p < .05 (2- tailed), **p < .01 (2-tailed). Sample size differs for different variables due to missing data and ranges from *N =* 135–293 for the total sample, *N* = 56–126 for boys, *N* = 79–167 for girls

**Cross-sectional relationships in total sample.** For the total sample at 7 years old, self-esteem was positively associated with body satisfaction while socially prescribed perfectionism, mother body dissatisfaction, and peer teasing were negatively correlated with body satisfaction. For the total sample at 8 years old, a positive association was identified between self-esteem and body satisfaction while negative relationships were found for anxiety/depression, socially prescribed perfectionism, and mother body dissatisfaction with (child) body satisfaction.

A multiple regression analysis was performed to assess the amount of variance in body satisfaction explained by self-esteem, socially prescribed perfectionism, mother body dissatisfaction, and peer teasing in the total sample at 7 years old. The model predicted 31.1% of the variance in children’s body satisfaction, *F* (4, 256) = 28.9, *p < .*001. Self-esteem, socially prescribed perfectionism, and mother body satisfaction were significant unique correlates of child body satisfaction.

A multiple regression analysis was performed to assess the amount of variance in body satisfaction explained by anxiety/depression, self-esteem, socially prescribed perfectionism, and mother body dissatisfaction in the total sample of 8-year-old children. The model predicted 25.7% of the variance in children’s body satisfaction, *F* (4, 196) = 17.00, *p* < .001. Self-esteem and mother body satisfaction were significant unique correlates of children’s body satisfaction.

**Table 3:** Multiple regression analysis for biopsychosocial variables cross-sectionally predicting body satisfaction at 7-Years-old for the total sample, boys, and girls

Body Satisfaction	B	SE_B_	β
Total Sample			
Child self-esteem Child socially prescribed perfectionism Mother body dissatisfaction Peer teasing	0.06 -0.02 -0.01 -0.02	0.01 0.01 0.00 0.01	0.46** -0.17** -0.14** -0.09
Boys			
hild self-esteem Child socially prescribed perfectionism Peer teasing	0.07 -0.02 -0.02	0.01 0.01 0.02	0.48** -0.22** -0.08
Girls			
Child self-esteem Child socially prescribed perfectionism Mother body dissatisfaction Peer teasing Child media exposure	0.07 -0.01 -0.01 -0.02 0.26	0.01 0.01 0.00 0.01 0.14	0.46** -0.09 -0.12 -0.11 -0.14

Note. ** *p* < .01, * *p* < .05; variables entered were those that were significant in Time 1 bivariate correlation analyses

**Table 4:** Multiple regression analysis for biopsychosocial variables cross-sectionally predicting body satisfaction at 8-years-old for the total sample, boys, and girls

Body Satisfaction	B	SE_B_	β
Total sample			
Child anxiety/depression Child self-esteem Child socially prescribed perfectionism Mother body dissatisfaction	0.00 -0.08 -0.00 -0.00	0.00 0.01 0.01 0.00	− 0.05 0.46** -0.03 -0.12*
Boys			
Child anxiety/depression Child self-esteem	0.00 -0.06	0.00 0.02	0.07 -0.27**
Girls			
Child anxiety/depression Child self-esteem Child socially prescribed perfectionism	0.00 -0.08 -0.01	0.00 0.01 0.01	0.08 -0.54** -0.08

Note. ** *p* < .01, * *p* < .05; variables entered were those that were significant in Time 2 bivariate correlation analyses

**Cross-sectional relationships for boys.** For 7-year-old boys, a positive association was identified between self-esteem and body satisfaction, and negative associations emerged for socially prescribed perfectionism and peer teasing with body satisfaction. At 8 years old for boys, greater levels of anxiety/depression were associated with lower body satisfaction and self-esteem was positively associated with body satisfaction.

A multiple regression analysis was performed to assess the amount of variance in body satisfaction explained by self-esteem, socially prescribed perfectionism, and peer teasing in boys at 7-years-old. The model predicted 30.8% of the variance in boys’ body satisfaction, *F* (3, 106) = 15.7, *p* < .001. Self-esteem and socially prescribed perfectionism were significant unique correlates of body satisfaction.

A multiple regression analysis was performed to assess the amount of variance in body satisfaction explained by anxiety/depression and self-esteem in 8-year-old boys. The model predicted 8.35% of the variance in boys’ body satisfaction, *F* (2, 84) = 5.51, *p* = .026. Self-esteem was a significant unique correlate of boys’ body satisfaction.

**Cross-sectional relationships for girls.** For 7-year-old girls, self-esteem was positively associated with body satisfaction while greater levels of socially prescribed perfectionism, greater frequency of peer teasing, greater exposure to media, and greater levels of mother body dissatisfaction were significantly associated with lower child body satisfaction. At 8 years old, self-esteem was positively associated with body satisfaction and greater levels of anxiety/depression and socially prescribed perfectionism were associated with lower body satisfaction.

A multiple regression analysis was performed to assess the amount of variance in child body satisfaction explained by child self-esteem, child socially prescribed perfectionism, mother body dissatisfaction, peer teasing, and child media exposure in 7-year-old girls. The model predicted 32.8% of the variance in girls’ body satisfaction, *F* (5, 145) = 14.20, *p* < .001. Self-esteem was the only significant unique correlate of body satisfaction.

A multiple regression analysis was performed to assess the amount of variance in body satisfaction explained by child anxiety/depression, child self-esteem, and child socially prescribed perfectionism in 8-year-old girls. The model predicted 34.2% of the variance in girls’ body satisfaction, *F* (3, 112) = 19.40, *p* < .001. Self-esteem was the only unique correlate of girls’ body satisfaction.

### Prospective relationships between biopsychosocial variables at 7-years-old and body satisfaction at 8-years-old

**Changes in body satisfaction over time.** Differences in mean scores for body satisfaction at Time 1 and Time 2 were explored using paired samples t-tests. At Time 1, the total sample had significantly lower scores for body satisfaction (*M* = 16.8, *SD* = 2.53) than at Time 2 (*M* = 17.3, *SD* = 2.20), *t*(215) = -2.55, *p* = .012. For girls, means for body satisfaction were directionally lower at Time 1 (*M* = 16.9, *SD* = 2.31) than at Time 2 (*M* = 17.2, *SD* = 2.37) but this was not significant, *t*(124) = -1.02, *p* = .308. For boys, body satisfaction was significantly lower at Time 1 (*M* = 16.7, *SD* = 2.82) than at Time 2 (*M* = 17.5, *SD* = 1.93), *t*(90) = -2.69, *p* = .009.

**Prospective relationships in the total sample.** Partial correlations for the total sample between biopsychosocial variables at Time 1 and body satisfaction at Time 2 after accounting for Time 1 body satisfaction revealed that greater child self-esteem at Time 1 predicted greater child body satisfaction at Time 2 after accounting for Time 1 child body satisfaction, and greater mother body dissatisfaction at Time 1 predicted lower child body satisfaction at Time 2. Higher BMI, greater anxiety/depression, and greater frequency of peer teasing at Time 1 showed trends (*p* < .10) towards predicting lower child body satisfaction at Time 2 after taking Time 1 child body satisfaction into account. Findings from partial correlations are presented in Table 5.

**Table 5:** Partial correlations between time 1 biopsychosocial variables and time 2 child body satisfaction for total sample, boys, and girls

	T2 Body Satisfaction		
	Total sample	Boys	Girls
Biopsychosocial Variable			
T1 Biological variables			
Child BMI ^δ^	− .13^δ^	− 0.11	− 0.15
T1 Psychological variables			
Child anxiety/depression ^δ^	− .13^δ^	− 0.15	− 0.09
Child self esteem ^δ^	0.14*	− 0.00	0.24**
Child self-oriented perfectionism	0.05	0.18	− 0.06
Child socially prescribed perfectionism ^δ^	− 0.01	0.09	− 0.13
T1 Sociocultural variables			
Mother body dissatisfaction	− 0.17*	− 0.22*	− 0.12
Mother comments	− 0.05	− 0.02	− 0.09
Father body dissatisfaction	− 0.08	− 0.09	− 0.08
Father comments	0.05	0.06	0.04
Peer teasing ^a^	− .15^δ^	− 0.11	− 0.05
Child media exposure	− 0.06	0.01	− 0.11

*Note*. Body dissatisfaction at Time 1 was partialled in these analyses

^a^ Spearman’s rho statistic used due to non-normal distribution of scores. ^δ^*p < .*10, *p < .05 (2- tailed), **p < .01 (2-tailed). Sample size differs for different variables due to missing data and ranges from *N =* 81–216 for the total sample, *N =* 36–91 for boys, *N =* 41–122 for girls

A hierarchical regression analysis was conducted to determine whether Time 1 child BMI, child anxiety/depression, child self-esteem, mother body satisfaction, and peer teasing were unique predicters of Time 2 child body satisfaction after Time 1 child body satisfaction was controlled. The results revealed that the model as a whole was significant, explaining 17.9% of the variance in Time 2 child body satisfaction, *F* (6, 189) = 6.85, *p < .*001. However, most of this variance was explained by Time 1 child body satisfaction and after this variable was controlled, Time 1 child BMI, child anxiety/depression, child self-esteem, mother body satisfaction, and peer teasing accounted for an additional 4.8% of the variance, which was not statistically significant, *F* change (5, 189) = 2.21, *p = .*055. Findings from hierarchical regressions are presented in Table 6.

**Table 6:** Hierarchical regression analysis for Time 1 Biopsychosocial Variables Predicting Time 2 child body satisfaction for the total sample, boys, and girls

Variable	B	SE_B_	β
Total sample			
Step 1			
Constant	0.18	0.03	
T1 Child body satisfaction	0.35	0.07	0.36**
Step 2			
Constant	0.45	0.32	
T1 Child body satisfaction	0.26	0.08	0.27**
T1 Child BMI	-0.02	0.01	-0.09
T1 Child anxiety/depression	-0.01	0.00	-0.09
T1 Child self-esteem	0.01	0.01	0.07
T1 Mother body dissatisfaction	-0.01	0.00	-0.12
T1 Peer teasing	-0.02	0.02	-0.10
Boys			
Step 1			
Constant	0.18	0.05	
T1 Child body satisfaction	0.31	0.09	0.34**
Step 2			
Constant	-0.06	0.12	
T1 Child body satisfaction	0.29	0.09	0.31**
T1 Mother body dissatisfaction	-0.01	0.01	-0.21*
Girls			
Step 1			
Constant	0.18	04	
T1 Child body satisfaction	0.42	0.08	0.42**
Step 2			
Constant	0.45	0.22	
T1 Child body satisfaction	0.36	0.09	0.36**
T1 Child self-esteem	0.02	0.01	0.12

Note. ** *p* < .01, * *p* < .05; variables entered were those that were significant in prospective partial correlation analyses

**Prospective relationships in boys.** Partial correlations were performed to investigate relationships between biopsychosocial variables at Time 1 and body satisfaction at Time 2 for boys. Findings indicate that greater mother body dissatisfaction at Time 1 was a significant partial correlate of lower child body satisfaction at Time 2 after Time 1 child body satisfaction was taken into account. A hierarchical regression was conducted to determine whether Time 1 mother body satisfaction predicted Time 2 child body satisfaction for boys after Time 1 child body satisfaction was controlled. The total variance in Time 2 child body satisfaction explained by the model as a whole was 15.5%, *F* (2, 87) = 7.99, *p < .*001. After controlling for Time 1 child body satisfaction, Time 1 mother body satisfaction accounted for an additional 4.3% of the variance in Time 2 child body satisfaction, *F* change (1, 87) = 4.40, *p = .*039. In the final model, mother body satisfaction was a significant unique predictor of change in body satisfaction for boys.

**Prospective relationships in girls.** A series of partial correlations were performed to investigate relationships between biopsychosocial variables at Time 1 and child body satisfaction at Time 2 for girls. Findings indicate that a greater child self-esteem at Time 1 was associated with greater child body satisfaction at Time 2 after controlling for Time 1 child body satisfaction. A hierarchical regression was conducted to determine whether Time 1 child self-esteem predicted Time 2 child body satisfaction for girls after Time 1 child body satisfaction was controlled. Results revealed that the model as a whole was significant, explaining 18.8% of the variance in Time 2 child body satisfaction, *F* (2, 122) = 14.10, *p < .*001. However, most of this variance was explained by Time 1 child body satisfaction and after this variable was controlled, Time 1 child self-esteem accounted for an additional 1.1% of the variance, which was not statistically significant, *F* change (1, 122) = 1.66, *p = .*20.

## Discussion

Although it is recognised that body image attitudes are frequently established in childhood, few studies have explored factors that may influence their development. The primary aim of this study was to identify key relationships between body satisfaction and biological, psychological, and sociocultural factors in 7- and 8-year-old girls and boys both cross-sectionally and longitudinally. In partial support for our hypotheses, multiple regression analyses revealed that child self-esteem and socially prescribed perfectionism, and mother body dissatisfaction in the total sample at 7-years, as well as child self-esteem and mother body dissatisfaction in the total sample at 8-years were significant unique cross-sectional correlates of child body satisfaction. Child self-esteem and socially prescribed perfectionism, and mother body dissatisfaction were the most relevant factors related to children’s body satisfaction, particularly cross-sectionally.

### Cross-sectional findings

Regarding biological factors, it is of interest that BMI was unrelated to body satisfaction in this sample when this relationship is consistently observed in adolescents [28, 83, 84]. A possible explanation for the absence of significant relationships is that perceived body size plays a more important role in determining 7- and 8-year-old children’s body satisfaction than actual body size. Previous research has revealed that there is frequently a discrepancy between children’s estimation of their body size and their actual body size, and that the capacity to accurately perceive body size increases with age, with girls acquiring accuracy earlier than boys at around age 10 to 12 years [85].

Cross-sectionally, as expected, greater self-esteem statistically predicted greater body satisfaction for the total sample and for boys and girls separately at both 7 and 8 years old. Consistent with Nichols et al. in the same sample but at an earlier age, these findings suggest that there is no sex difference in the relationship between self-esteem and body satisfaction [12]. A possible explanation for the role of self-esteem in body satisfaction is that children who have a greater level of regard for their worth may be more resilient in response to external appearance pressures. Conversely, children with lower self-esteem may be at greater risk of internalising societal appearance ideals and accepting these as being central to their worth [8]. The current findings may suggest that children with greater self-esteem are less likely to perceive their worth as being contingent upon meeting societal expectations. Importantly, temporal precedence cannot be determined solely based on cross-sectional findings.

Socially prescribed perfectionism was found to be negatively correlated with body satisfaction for the total sample and girls at age 7 and 8, and for boys at age 7. In cross-sectional multivariate analyses, socially prescribed perfectionism was statistically correlated with 7-year-old boys’ body satisfaction; however, for 7- and 8-year-old girls, socially prescribed perfectionism was not a significant unique correlate when considered in the context of a combination of biopsychosocial variables. For girls, self-esteem is identified as more strongly related to body satisfaction. There were no significant relationships identified between self-oriented perfectionism and body satisfaction in this age group. It is possible that self-oriented perfectionism may become increasingly important later when perceived pressures from external sources are internalised and become expectations imposed on the self.

Cross-sectional negative correlations were identified between anxiety/depression and body satisfaction at 8 years old for the total sample, and for boys and girls. Consistent with Nichols et al. in the same sample but at an earlier age, these findings may suggest that children who experience higher levels of negative affect may be less inclined to perceive positive rewards from any source, including from the pursuit of societal appearance ideals [12]. However, it is important to note that this pattern was not found in 7-year-old children, and for most children in the current sample, scores for anxiety/depression fell within the normal range as reported by parents. Anxiety/depression was not a significant unique correlate of body satisfaction cross-sectionally in the context of a multivariate biopsychosocial model. It is unlikely that, if most children at this age are making overly positive evaluations of themselves, they would be associating negative mood states with critical self-evaluations regarding their appearance. Regardless, clinical level anxiety and depression has been reported among children as young as 4 years old [86, 87]; therefore, relationships with body satisfaction should be further investigated with larger samples of children with a wider variation in internalising symptoms to further understand the current findings.

In support of cross-sectional hypotheses, greater mother body dissatisfaction was correlated with lower body satisfaction for the total sample at 7 and 8 years old and for 7-year-old girls. In cross-sectional multivariate analyses, mother body dissatisfaction statistically predicted body satisfaction for the total sample at 7 and 8 years old. Most children in the current sample spent more time with their mother as their primary caregiver; therefore, it is plausible that there would be greater opportunity for mothers than fathers to express dissatisfaction with their own body in the presence of their child. Contrary to hypotheses, parent comments were not significantly related to children’s body satisfaction cross-sectionally which may be related to the low prevalence of comments from both mothers and fathers in the current sample.

Hypotheses for the relationship between peer teasing and body satisfaction were partially supported. Cross-sectionally, greater frequency of peer teasing was significantly correlated with lower body satisfaction for the total sample, boys, and girls at age 7 but not age 8. The Time 1 findings are consistent with previous studies including Day et al. where, in a systematic review involving children and adolescents, weight-related victimisation from peers was associated with body image disturbance, particularly among cross-sectional studies [57].

In partial support of hypotheses, a significant cross-sectional relationship was identified between media exposure and body satisfaction for 7-year-old girls, and this is consistent with studies involving 5- to 8-year-old girls [10, 19]. However, media exposure was not a significant cross-sectional correlate of body satisfaction for girls when considered with other biopsychosocial variables, suggesting that other factors, particularly self-esteem, may be more important. Additionally, there were no longitudinal relationships found for media exposure. It is important to consider limitations of the measure used to assess media in the current study when interpreting these findings. Specifically, media was measured based on parent’s estimation of the daily average number of hours that their child spends watching television, DVDs, online videos (e.g., YouTube), and playing computer games. The type of content consumed by children was not measured in this study and, therefore, it is not known whether children were exposed to appearance focused media or more general content. As children approach adolescence and the use of media, particularly social media, increases [88], relationships with body satisfaction may change. Therefore, relationships between media exposure and body satisfaction in children should be investigated further. Future research involving children could utilise a measure that is more specific to consumption of appearance focused media rather than time spent.

### Prospective findings

Biopsychosocial variables at age 7 were examined as prospective predictors of body satisfaction at age 8, after accounting for Time 1 body satisfaction. Between ages 7 and 8, body satisfaction showed some consistency over time, with Time 1 body satisfaction accounting for 17.7% of the variance for girls’ and 11.2% for boys’ Time 2 body satisfaction. This is consistent with studies of 11- to 12-year-old children [4, 5]. The only other significant prospective relationship found was that mother self-reported body dissatisfaction at child age 7 predicted boys’ body satisfaction at age 8 accounting for a further 4.3% of the variance explained. This suggests that greater mother body dissatisfaction predicted decreases in boys’ own body satisfaction over time. This was not replicated for girls, for whom there was only a non-significant tendency for child self-esteem at age 7 to predict body satisfaction at age 8. Regarding this finding, it is possible that mothers have more influence on boys’ body satisfaction while for girls, there may be other factors not explored in this study that are more relevant. Relationships between parents’ modelling of body image concerns and child body satisfaction are also unclear in adolescent samples [28, 30, 50]. More research with both children and adolescents would be needed to further interpret these findings.

Regarding non-significant findings, it is possible that prospective predictors of body satisfaction in young children have yet to be identified. However, it is also possible that the time frame between each assessment and ages of children may not have been optimal for finding prospective relationships, which is a challenge for all longitudinal research. There may be key developmental time points in which body satisfaction reduces and the period of time between 7 and 8 years old appears not to have been one of them, particularly for boys, as body satisfaction actually increased. Alternatively, while adequate internal consistency was found for most measures, children’s cognitive capacity for self-reflection may not be well established by ages 7 and 8, so accuracy of reporting or meta-cognitive ability may be less than in older children. Similarly, mother’s reports of children experiencing teasing may not be fully informed, as children may not report it to them when they experience it. Finally, it is possible based on findings pertaining to self-esteem in this study and in previous studies involving children (e.g., 11,12) that self-esteem and body satisfaction develop simultaneously in middle childhood and that a predictive relationship between these variables may emerge later. However, prospective studies involving adolescents have produced inconsistent findings regarding whether a significant relationship exists between self-esteem and body satisfaction [28–30]; therefore, further investigation is required.

Collectively, the current findings highlight that self-esteem, socially prescribed perfectionism, peer teasing, and mother body dissatisfaction are the most salient factors related to children’s body satisfaction, particularly cross-sectionally. Importantly, however, some relationships found in the current research were weak and others failed to reach significance which may be explained by the stage of cognitive development at 7 and 8 years old, especially since significant relationships have been found in older age groups. This may indicate an opportunity for future research to consider cognitive maturation in order to measure and interpret relationships between biopsychosocial variables and body satisfaction in children most accurately. Additionally, given that few significant prospective relationships were found in the current study and cross-sectional findings suggest, in some cases, co-development of variables, investigation of change over significant developmental periods, particularly between middle childhood and early adolescence, may be important for identifying prospective relationships.

As with all research, it is also important to consider limitations. Although examination of correlates and prospective predictors of body satisfaction in 8-year-old children was the primary purpose of this research, the study and analytic approach were not pre-registered. Bias should be considered as it is likely that children of parents who were interested in body image were overrepresented in the current sample given parents’ awareness that the research was investigating risk factors involved in the development of body satisfaction. In addition, the sample was not large, and corrections have not been made for the number of variables in analyses so caution is needed when interpreting results. A further limitation is that the study was conducted with children from a Western culture, most children were from middle to upper socioeconomic backgrounds, and parents of children were generally more highly educated than average Australian samples. Consequently, findings may not be generalisable to other demographic groups.

## Conclusions

This is the first study to investigate relationships between a range of biopsychosocial factors and body satisfaction in 7- and 8-year-old children both cross-sectionally and longitudinally. Overall, self-esteem, socially prescribed perfectionism, mother body dissatisfaction, and peer teasing were the key factors related to children’s body satisfaction, particularly cross-sectionally. Child body satisfaction at age 7 significantly predicted child body satisfaction at age 8, suggesting the potential stability of body satisfaction in children over time. The current findings suggest co-development of variables with body satisfaction and suggest opportunities for future research to investigate change in relationships between the period from middle childhood to early adolescence. Further research is needed to replicate the findings, particularly with larger samples to support the conclusions from this study.

## Data Availability

No datasets were generated or analysed during the current study.
